# The new oncogene transmembrane protein 60 is a potential therapeutic target in glioma

**DOI:** 10.3389/fgene.2022.1029270

**Published:** 2023-01-20

**Authors:** Fengdong Yang, Xuezhi Zhang, Xinzhuang Wang, Yake Xue, Xianzhi Liu

**Affiliations:** Department of Neurosurgery, First Affiliated Hospital of Zhengzhou University, Zhengzhou, Henan Province, China

**Keywords:** transmembrane protein 60, isocitrate dehydrogenase 1, 1p19q, glioma, biomarker, tumor microenvironment

## Abstract

Glioma is a malignant tumor with a high fatality rate, originating in the central nervous system. Even after standard treatment, the prognosis remains unsatisfactory, probably due to the lack of effective therapeutic targets. The family of transmembrane proteins (TMEM) is a large family of genes that encode proteins closely related to the malicious behavior of tumors. Thus, it is necessary to explore the molecular and clinical characteristics of newly identified oncogenes, such as transmembrane protein 60 (TMEM60), to develop effective treating options for glioma. We used bioinformatic methods and basic experiments to verify the expression of transmembrane protein 60 in gliomas and its relationship with 1p and 19q (1p19q) status, isocitrate dehydrogenase (IDH) status, patient prognosis, and immune cell infiltration using public databases and clinical samples. In addition, Gene Ontology (GO) and Kyoto Encyclopedia of Genes and Genomes (KEGG) enrichment analyses were performed to detect co-expressed genes. Thus, we inhibited the expression of transmembrane protein 60 to observe the proliferation and activity of glioma LN229 cells. We found transmembrane protein 60 was significantly upregulated in glioma compared with that in normal brain tissue at the mRNA. In the subgroups of World Health Organization high grade, isocitrate dehydrogenase wildtype, 1p and 19q non-codeletion, or isocitrate dehydrogenase wild combined with 1p and 19q non-codeletion, the expression of transmembrane protein 60 increased, and the prognosis of glioma patients worsened. In the transmembrane protein 60 high expression group, infiltration of immune cells and stromal cells in the tumor microenvironment increased, tumor purity decreased, and immune cells and pathways were activated. The immune cells mainly included regulatory T-cell, gamma delta T-cell, macrophages M0, neutrophils, and CD8^+^ T-cells. Overexpression of co-inhibitory receptors (CTLA4, PDL1 and CD96) may promote the increase of depletion of T-cell, thus losing the anti-tumor function in the transmembrane protein 60 high expression group. Finally, we found that transmembrane protein 60 silencing weakened the viability, proliferation, and colony formation of glioma LN229 cells. This is the 0 report on the abnormally high expression of transmembrane protein 60 in glioma and its related clinical features, such as tumor microenvironment, immune response, tumor heterogeneity, and patient prognosis. We also found that transmembrane protein 60 silencing weakened the proliferation and colony formation of glioma LN229 cells. Thus, the new oncogene transmembrane protein 60 might be an effective therapeutic target for the clinical treatment of glioma.

## Introduction

Primary brain tumors (e.g., benign tumors and malicious tumors) are highly heterogeneous and originate from cells of the central nervous system. Gliomas are mainly occurred in the supra-tentorium (frontal, temporal, parietal, and occipital lobes combined) (61.4%). Only a very small proportion occurred in areas of the CNS other than the brain (Ostrom et al., 2020; Ostrom et al., 2021). Currently, the overall median survival of glioma patients is less than 2 years, even after standard treatment (Xu et al., 2021; Zeng et al., 2021). Some new clinical methods were applied but with no significant effects, revealing the lack of effective therapeutic targets (Mercer et al., 2009; Gusyatiner and Hegi, 2018; Lim et al., 2018). Chimeric antigen receptor T-cell (CAR-T) therapy used in treating hematologic malignancies might be a promising method for glioma patients (Labanieh et al., 2018); thus, it is crucial to identify oncogenes that can be used as therapeutic targets.

With the development of sequencing technology and the free access to public databases, many new biomarkers have been screened and applied clinically (Pallen et al., 2010; Chen et al., 2017a). For instance, isocitrate dehydrogenase (IDH) mutations often occur in low-grade gliomas, and the overall prognosis of patients with IDH mutations is better than that of wild-type patients (Cohen et al., 2013; Reuss et al., 2015). Patients with combined deletion of 1p and 19q (1p19q) are usually sensitive to chemotherapy drugs and can guide clinical treatment (Cancer Genome Atlas Research Network Brat et al., 2015; Eckel-Passow et al., 2015), while epidermal growth factor receptor (EGFR) has shown to possess carcinogenic properties in various tumors, and targeted molecular therapy for EGFR has achieved good results (Felsberg et al., 2017). Nevertheless, the existing molecular markers only target specific populations without elucidating the pathological mechanism of glioma (Pitt et al., 2016; Nørøxe et al., 2017). Thus, new molecular markers are urgently needed to improve the prognosis of patients and identify new therapeutic targets.

The family of transmembrane proteins (TMEM) is a large family of genes that encode proteins closely related to the malicious behavior of tumors. For example, TMEM45A can attenuate the killing effect of drugs on glioma cells and promote malignant proliferation (Sun et al., 2015); TMEM88 promotes breast cancer invasion and migration by interacting with dishevelled (Dvl) proteins (Yu et al., 2015); TMEM45B promotes limitless proliferation of gastric cancer cells through JAK2/STAT3 (Shen et al., 2018); TMEM60 increases marbling fat in beef through the co-expression network (Lim et al., 2014) and, when combined with other genes, participates in the production and secretion of creatinine in some European bloodlines (Liu et al., 2011).

Our study showed that TMEM60 is abnormally highly expressed in gliomas using data from multiple centers, populations, and clinical samples. Its abnormal expression is closely related to patient prognosis, IDH wild-type status, 1p19q non-codeletion status, tumor immune microenvironment, tumor burden-related factors, and immune cell infiltration. Therefore, TMEM60 might be a molecular marker that could be used as a new therapeutic target for glioma treatment.

## Materials and methods

### Clinical samples

All glioma tissue samples were obtained from the surgical resection of tumors from glioma patients (n = 30). Non-tumor brain tissue was used as the negative control group (n = 5). The tissue samples were stored separately in liquid nitrogen. This study was approved by the ethics committee of the First Affiliated Hospital of Zhengzhou University. Written informed consent was obtained from all patients.

### Data extraction

Data were downloaded from The Cancer Genome Atlas (TCGA) (https://www.cancer.gov), the Chinese Glioma Genome Atlas (CGGA) (http://www.cgga.org.cn), and the Gene Expression Omnibus (GEO) (https://www.ncbi.nlm.nih.gov). After excluding samples with incomplete clinical information, we collected 546 samples from TCGA RNA sequencing (RNA-seq), 749 samples from CGGA RNA-seq (Bao et al., 2014), and 268 samples from CGGA Array (Fang et al., 2017; Liu et al., 2018). In addition, we downloaded 42 samples from GSE116520 (Kruthika et al., 2019) and six samples from GSE153692. The clinical information of each sample included age, glioma grade, IDH mutation status, 1p19q deletion status, and survival information. The expression data of TMEM60 in different tumors, the somatic copy number alterations (SCNA) module of TMEM60, and the immune cell infiltration of different TMEM60 expression groups were obtained from the TIMRE database.

### Data analysis

Wilcoxon test was used to identify differences in TMEM60 expression between the glioma and normal brain tissues. Wilcoxon test or Kruskal-Wallis test was used to analyze the relationship between clinical symptoms and TMEM60 expression. Survival and receiver operating characteristic (ROC) curves were illustrated using the packages “survival” and “survival ROC” in R. The packages “GSVA,” “limma,” and “GSEABase” in R were used to score immune gene sets in tumor samples. Heat maps of immune and clinical symptoms were illustrated using the package ‘heatmap’ in R. The infiltration of immune cells in tumor samples was calculated using CIBERSORT R script 1.03 (Newman et al., 2015). The differences in immune cell infiltration between the high and low expression groups were plotted with the package “Vioplot” in R using the median as the cutoff value.

### Quantitative reverse transcription PCR (qRT‐PCR)

Total RNA was extracted using TRIzol reagent (Cat# 15596026, Invitrogen, United States) according to the manufacturer’s specifications. Total RNA concentration was detected using NanoDrop 2000 (Thermo Scientific, Waltham, MA, United States). Total RNA was reverse transcribed using a qPCR RT Kit (Cat# FSQ-101, Toyobo, Osaka, Japan) with 37 C for 15 min, then 98 C for 5 min. The relative expression of the target gene was determined using qRT-PCR with FastStart universal 96 SYBR Green Master (Cat# 4913914001, Roche, Germany) and GAPDH as the housekeeping gene. The primer sequences used in this experiment are as follows: TMEM60(NM_032936.4), 5′-GTC​CTG​CTG​ATT​GTG​AAA​ATG​GC-3′ and 5′-TGATCCATGTCG AGGGTCAAA-3′; GAPDH(NM_001256799), 5′-AAT​CCC​ATC​ACC​ATC​TTC-3′ and 5′-AGGCTG TTGTCATACTT C-3′. The quantification of genes was displayed by the ∆∆Ct method [∆∆Ct = ∆Ct (sample)−∆Ct (control average)].

### Enrichment analysis

Differential genes co-expressed with TMEM60 (cor >.5) in tumor samples from the TCGA and CGGA databases were screened using the “cor.test” function in R, and the intersection was further identified through the Venn diagram. Functional analysis of differentially expressed genes was performed using the packages “clusterProfiler,” “org.Hs.eg.db,” “enrichplot,” and “ggplot2” in R.

### Cell viability and clone formation assay

Human glioblastoma cell lines (LN229, U251, T98, U87, and A172) and normal human astrocytes (NHA) were purchased from Wuhan Prosei Life Technology (Wuhan, Hubei, China). Glioblastoma (GBM) cells were seeded in a 96-well plate at a density of 5 ×10^3^ cells/well. After 24 h, si-TMEM60 was transfected into the cells using Lipofectamine 2000. The absorbance values at 0 h, 24 h, 48 h, and 72 h post-transfections were detected by 3-(4,5-dimethylthiazol-2-yl)-2,5-diphenyl-2H-tetrazolium bromide (MTT; Cat# HY-15924, MedChemExpress, United States). GBM cells were seeded in a six-well plate at a density of 5 ×10^2^ cells/well and observed for 2 weeks.

### Immunofluorescence staining

A total of 1×10^5^ LN229 cells were treated with si-TMEM60, fixed with 4% paraformaldehyde, permeabilized with Triton X-100, blocked with 10% bovine serum albumin, and incubated with the Ki67 primary antibody (Cat# AF0198, affinity, United States) overnight at 4 C. Florescent secondary antibody (Cat#ab6046, Abcam, UK) and DAPI (Cat# C0060, Solarbio, China) were used for fluorescence microscopy the following day.

### Statistical analysis

R x64 3.5.2, Strawberry-Perl-5.30.2.1, and GraphPad Prism7 were used for statistical analyses and graphical representations. Two or more groups was performed using Wilcoxon or kruskal. test, and statistical significance was set at *p* < .05. The data was presented as mean and SD in this paper.

## Results

### Transmembrane protein 60 is associated with poor prognosis in glioma patients

**FIGURE 1:**
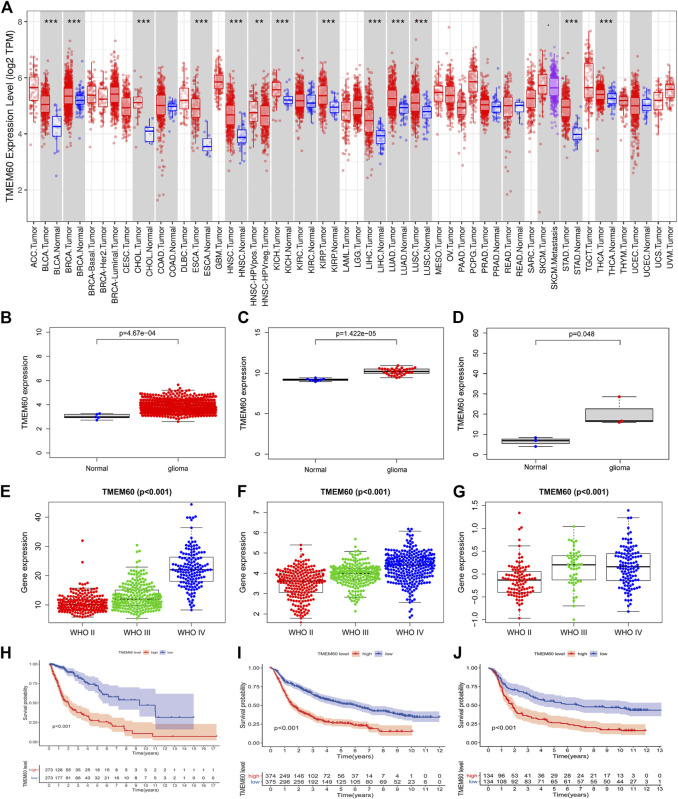
High expression of transmembrane protein 60 (TMEM60) indicates poor prognosis in glioma patients. **(A)** Relative expression of TMEM60 in various tumors based on the TIMRE database. Expression levels of TMEM60 in all gliomas and normal brain tissues based on **(B)** The Cancer Genome Atlas (TCGA), **(C)** GSE116520, and **(D)** GSE153692. Relationship between the expression levels of TMEM60 and the World Health Organization (WHO) grades based on **(E)** TCGA mRNA sequencing (mRNA-seq), **(F)** Chinese Glioma Genome Atlas (CGGA) mRNA-seq, and **(G)** CGGA microarray. Survival curve of glioma patients related to TMEM60 expression based on **(H)** TCGA mRNA-seq, **(I)** CGGA mRNA-seq, and **(J)** CGGA microarray.

**FIGURE 2:**
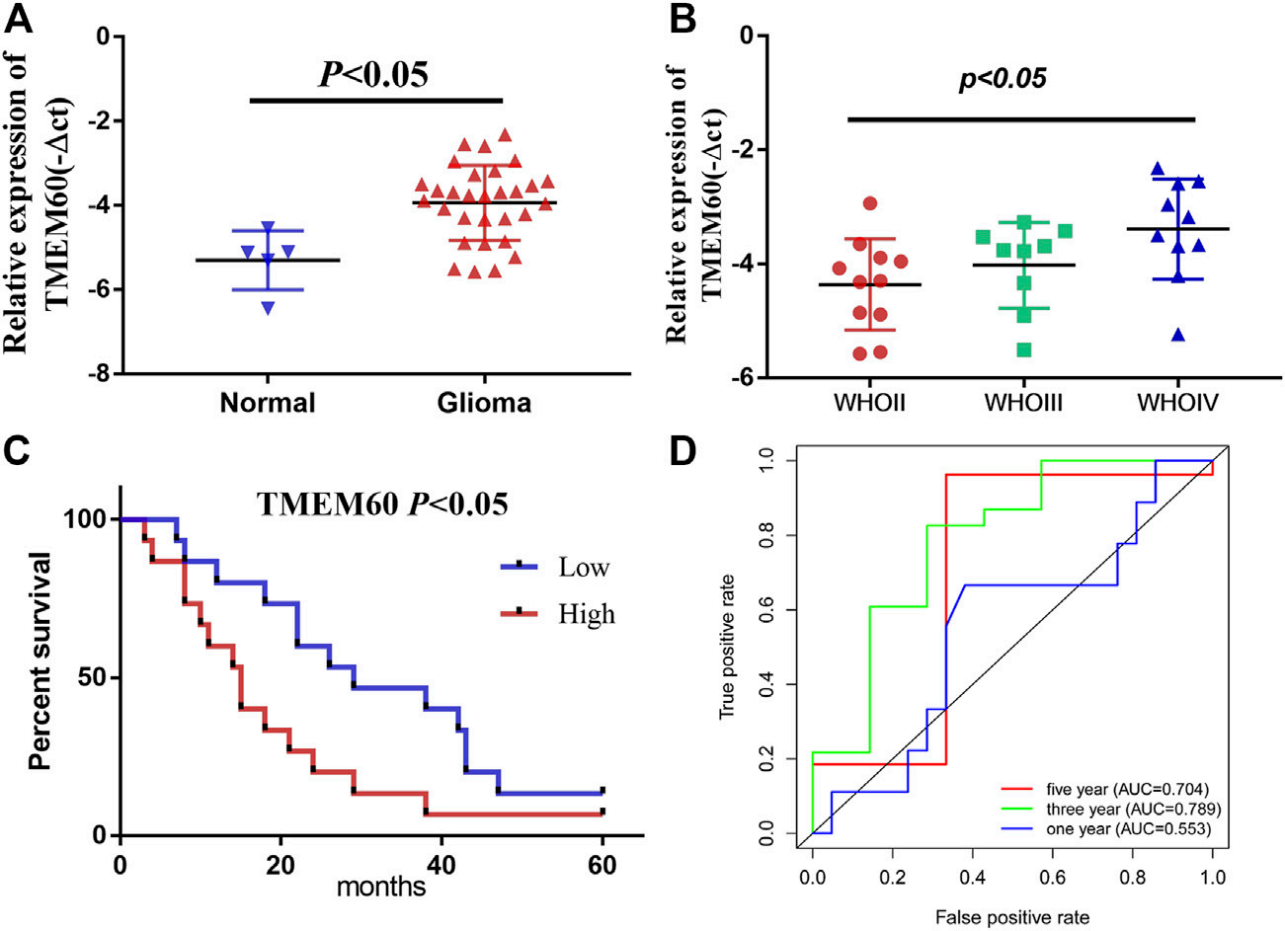
Relative expression and clinical significance of TMEM60 in clinical samples. **(A)** TMEM60 expression in different tissues and **(B)** different WHO grades. **(C)** Correlation between TMEM60 expression and prognosis of glioma patients. **(D)** Receiver operating characteristic (ROC) curve of TMEM60 and prognosis of glioma patients.

Using the TIMRE database, we found that TMEM60 was abnormally expressed in various tumors (Figure 1A). In addition, TMEM60 was significantly upregulated in glioma compared with normal brain tissue from TCGA (Figure 1B; *p* < .001), GSE116520 (Figure 1C; *p* < .001), and GSE153692 (Figure 1D; *p < .05*), However, there are no meaningful results in the CGGA database. These results were verified using clinical samples (Figure 2A; *p* < .05). We also found that the World Health Organization (WHO) grade was positively correlated with the expression of TMEM60 in glioma using data from TCGA RNA-seq (Figure 1E; *p* < .001), CGGA RNA-seq (Figure 1F; *p* < .001), CGGA mRNA-array (Figure 1G; *p* < .001), and 30 clinical glioma samples (Figure 2B; *p* < .05). Survival analysis showed that patients in the high expression group had worse prognosis than those in the low expression group of TCGA RNA-seq (Figure 1H; *p* < .001), CGGA RNA-seq (Figure 1I; *p* < .001), CGGA mRNA-array (Figure 1J; *p* < .001), and clinical samples (Figure 2C; *p* < .05).

### Transmembrane protein 60 is an independent prognostic factor for glioma patients

**FIGURE 3:**
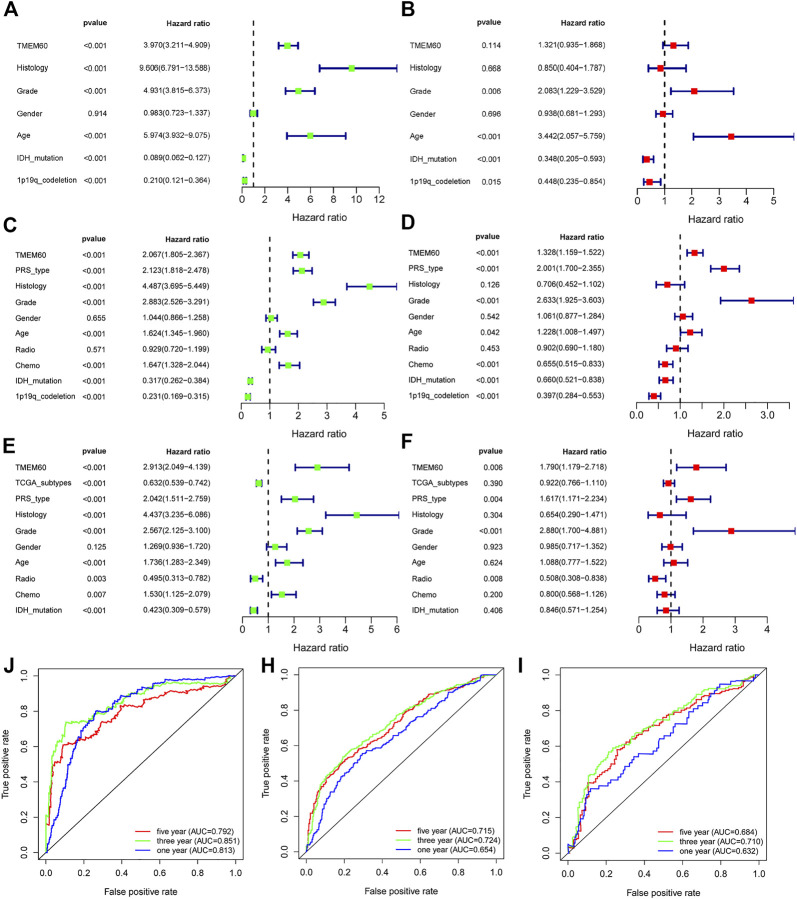
TMEM60 is an independent prognostic factor for glioma patients. Univariate analysis, multivariate analysis, and ROC curve analysis related to clinical indicators of glioma patients and TMEM60 expression in **(A,B and J)** TCGA, **(C,D and H)** CGGA-325, and **(E,F and I)** CGGA-693.

We conducted univariate survival analysis of clinical samples and found that TMEM60, age, and the WHO grade were risk factors closely related to prognosis (hazard ratio,HR > 1, *p* < .05). Nevertheless, HR was not statistically significant in factors such as sex, KPS score, tumor location, tumor volume, radiotherapy, or chemotherapy (Table 1). We further expanded the clinical sample information through the database to determine the potential application value. Based on univariate analysis, TMEM60 was found to be a risk factor for the prognosis of glioma patients based on TCGA RNA-seq (Figure 3A; HR = 3.970, 95% confidence interval (CI) = 3.211–4.909, *p* < .001), CGGA RNA-seq (Figure 3C; HR = 2.067, 95% CI = 1.805–2.367, *p* < .001), and CGGA RNA-array (Figure 3E; HR = 2.913, 95% CI = 2.049–4.139, *p* < .001). Based on multivariate analysis, TMEM60 could be considered an independent risk factor for the prognosis of glioma patients based on CGGA RNA-seq (Figure 3D; HR = 1.328, 95% CI = 1.159–1.522, *p* < .001) and TGGA RNA-array (Figure 3F; HR = 1.790, 95% CI = 1.179–2.718, *p* < .01) but not based on TGGA RNA-seq (Figure 3B; HR = 1.321, 95% CI = .935–1.868, NS). Using the ROC curve, we found that TMEM60 was an independent prognostic factor for patients’ 3-year and 5-year survival rates based on TCGA RNA-seq (Figure 3J; area under the curve (AUC)_3year_ = .851, AUC_5year_ = .792), CGGA RNA-seq (Figure 3H; AUC_3year_ = .724, AUC_5year_ = .715), and CGGA RNA-array (Figure 3I; AUC_3year_ = .710, AUC_5year_ = .684), but not based on the clinical samples (Figure 2D; AUC_3year_ = .789, AUC_5year_ = .704).

**TABLE 1:** Association of TMEM60 expression is with the clinicopathological characteristics of patients with glioma.

Clinical Features	Patient number	TMEM60 expression				Univariate analysis		
		High	Low	χ2	P	HR	95%CI	*p*-value
Age				0.14	0.71	2.50	(1.07–5.79)	0.03
<48	13.00	6.00	7.00					
≥48	17.00	9.00	8.00					
Gender				0.13	0.72	1.47	(.69–3.14)	0.32
Female	15.00	8.00	7.00					
Male	15.00	7.00	8.00					
WHO grade				8.17	0.02	1.85	(1.09–3.13)	0.02
Ⅱ	11.00	2.00	9.00					
Ⅲ	9.00	5.00	4.00					
Ⅳ	10.00	8.00	2.00					
KPS score				2.40	0.12	1.88	(.87–4.08)	0.11
<80	10.00	7.00	3.00					
≥80	20.00	8.00	12.00					
Tumor location				0.69	0.87	1.29	(.93–1.79)	0.12
Occipital lobe	2.00	1.00	1.00					
Parietal lobe	3.00	2.00	1.00					
Temporal lobe	9.00	5.00	4.00					
Frontal lobe	16.00	7.00	9.00					
Tumor volume(cm3)				0.54	0.46	1.27	(.59–2.75)	0.54
<32	16.00	7.00	9.00					
≥32	14.00	8.00	6.00					
Postsurgical radiotherapy				0.14	0.70	0.72	(.33–1.59)	0.42
Yes	19.00	9.00	10.00					
No	11.00	6.00	5.00					
Postsurgical TMZ therapy				0.54	0.46	0.64	(.29–1.44)	0.28
Yes	14.00	8.00	6.00					
No	16.00	7.00	9.00					
TMEM60	30.00	15.00	15.00	-	-	1.57	(1.01–2.43)	0.04

Values in bold indicate statistical significance (*p* < .05). KPS, score, Karnofsky performance score; HR, hazard ratio; CI, confidence interval.

### Correlation between transmembrane protein 60 and isocitrate dehydrogenase status

**FIGURE 4:**
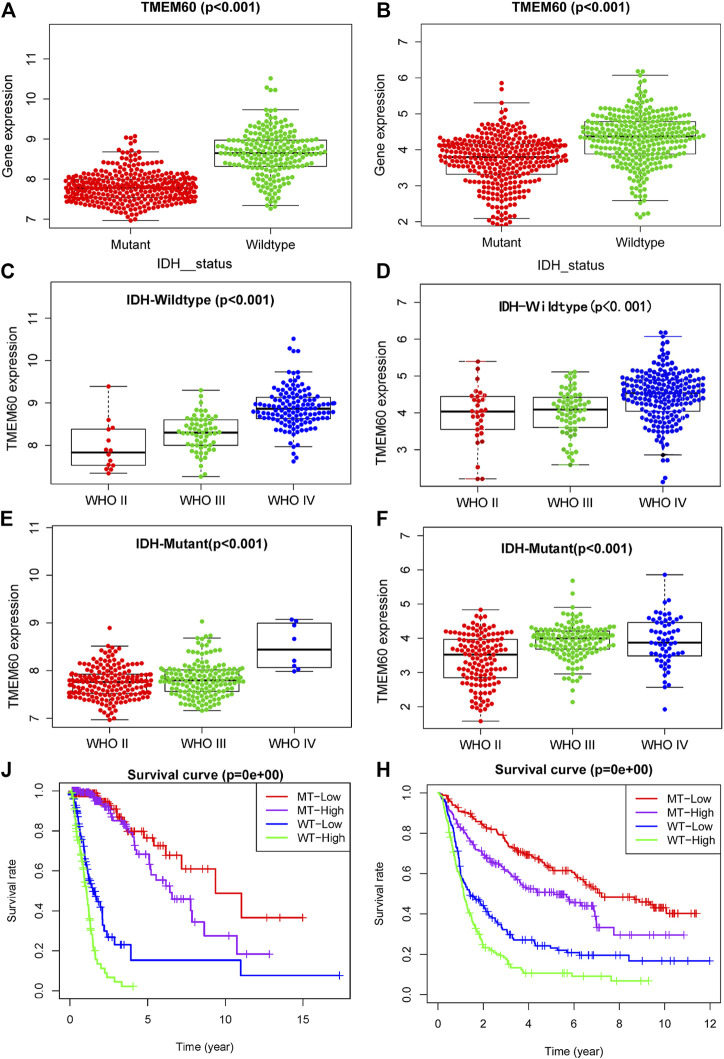
Effect of TMEM60 expression combined with isocitrate dehydrogenase (IDH) status on the survival time of glioma patients. Relationship between TMEM60 expression and IDH status based on **(A)** TCGA and **(B)** CGGA. Relationship between TMEM60 expression and the WHO grade in the subgroups of IDH mutants in **(C)** TCGA and **(D)** CGGA and the subgroups of IDH wild type in **(E)** TCGA and **(F)** CGGA. Relationship between TMEM60 expression and patient survival time in the subgroups of different IDH status in **(J)** TCGA and **(H)** CGGA. MT-low: IDH mutant and TMEM60 low expression; MT-High: IDH mutant and TMEM60 High expression; WT-low: IDH wildtype and TMEM60 low expression; WT-High: IDH wildtype and TMEM60 high expression.

IDH status, as a biomarker for the prognosis of glioma, has been clinically applied, but its relationship with TMEM60 remains unclear. Our analysis showed that the expression of TMEM60 in the IDH wild-type glioma subtype was higher than that in the IDH mutant subtype of TCGA (Figure 4A; *p* < .001) and CGGA (Figure 4B; *p* < .001). TMEM60 expression levels in the IDH wild-type glioma subgroup gradually increased with the higher WHO grade in TCGA (Figure 4C; *p* < .001) and CGGA (Figure 4D; *p* < .001). In addition, TMEM60 expression was significantly upregulated in high-grade IDH mutant glioma patients of TCGA (Figure 4E; *p* < .001) and CGGA (Figure 4F; *p* < .001). Survival analysis in TCGA (Figure 4J; *p* < .001) and CGGA (Figure 4H; *p* < .001) revealed that IDH wild-type patients with high TMEM60 expression levels had the worst prognosis, followed by IDH wild-type patients with low TMEM60 expression levels and IDH mutant patients with high TMEM60 expression levels. IDH mutant patients with low TMEM60 expression levels had the best prognosis.

### Correlation between transmembrane protein 60 and 1p and 19q status

**FIGURE 5:**
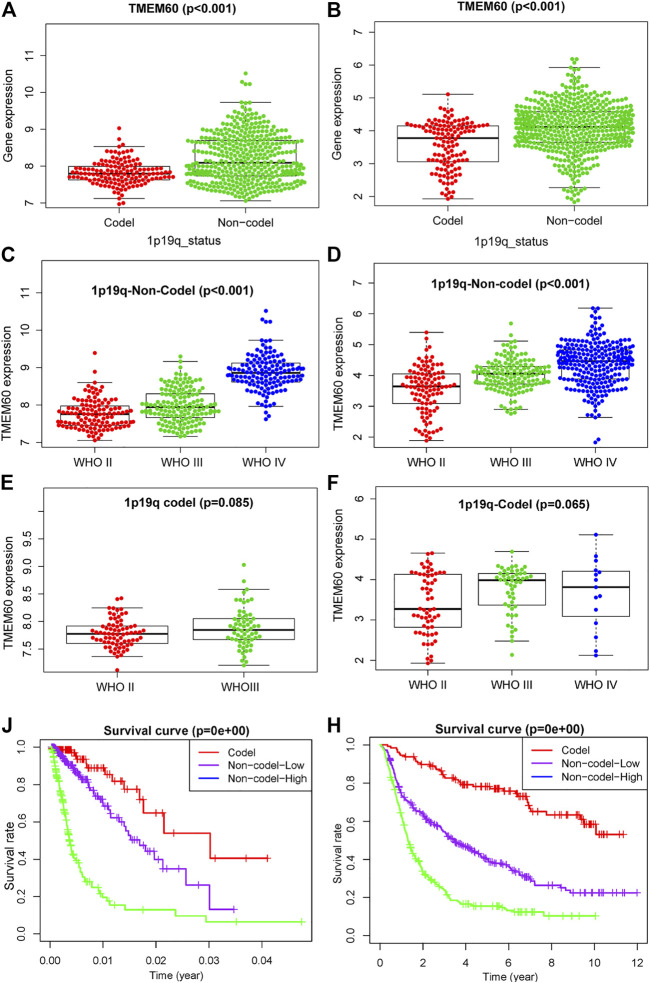
Effect of TMEM60 expression combined with 1p19q status on the survival time of glioma patients. Relationship between TMEM60 expression and 1p19q status in **(A)** TCGA and **(B)** CGGA. Relationship between TMEM60 expression and the WHO grade in the subgroups of 1p19q non-codel in **(C)** TCGA and **(D)** CGGA and the subgroups of 1p19q codel in **(E)** TCGA and **(F)** CGGA. Relationship between TMEM60 expression and patient survival time in the subgroups of different 1p19q status in **(J)** TCGA and **(H)** CGGA. Codel: 1p19q codeletion, Non-codel-Low: 1p19q non-codeletion and TMEM60 low expression, Non-codel-High:1p19q non-codeletion and TMEM60 high expression.

1p19q is often combined with IDH as a biomarker to distinguish different glioma subtypes. We found that the expression of IDH and TMEM60 had a synergistic effect and further explored the relationship between 1p19q and TMEM60 expression. In TCGA and CGGA, the expression of TMEM60 in the 1p19q non-codel subgroup was significantly upregulated compared with that in the 1p19q codel subgroup (Figures 5A,B). In the 1p19q non-codel subgroup, the upregulation of TMEM60 expression was accompanied by an increase in the WHO grade of TCGA (Figure 5C; *p* < .001) and CGGA (Figure 5D; *p* < .001). In the 1p19q codel subgroup, the results were insignificant in TCGA and CGGA (Figures 5E,F). Survival analysis in TCGA (Figure 5J; *p* < .001) and CGGA (Figure 5H; *p* < .001) revealed that 1p19q non-codel patients with high TMEM60 expression levels had the worst prognosis, followed by 1p19q non-codel patients with low TMEM60 expression levels and 1p19q codel patients (the high TMEM60 expression levels vs low TMEM60 expression levels were insignificant in 1p19q codel subgroup) had the best prognosis.

### Effect of transmembrane protein 60 combined with isocitrate dehydrogenase and 1p and 19q on glioma patients

**FIGURE 6:**
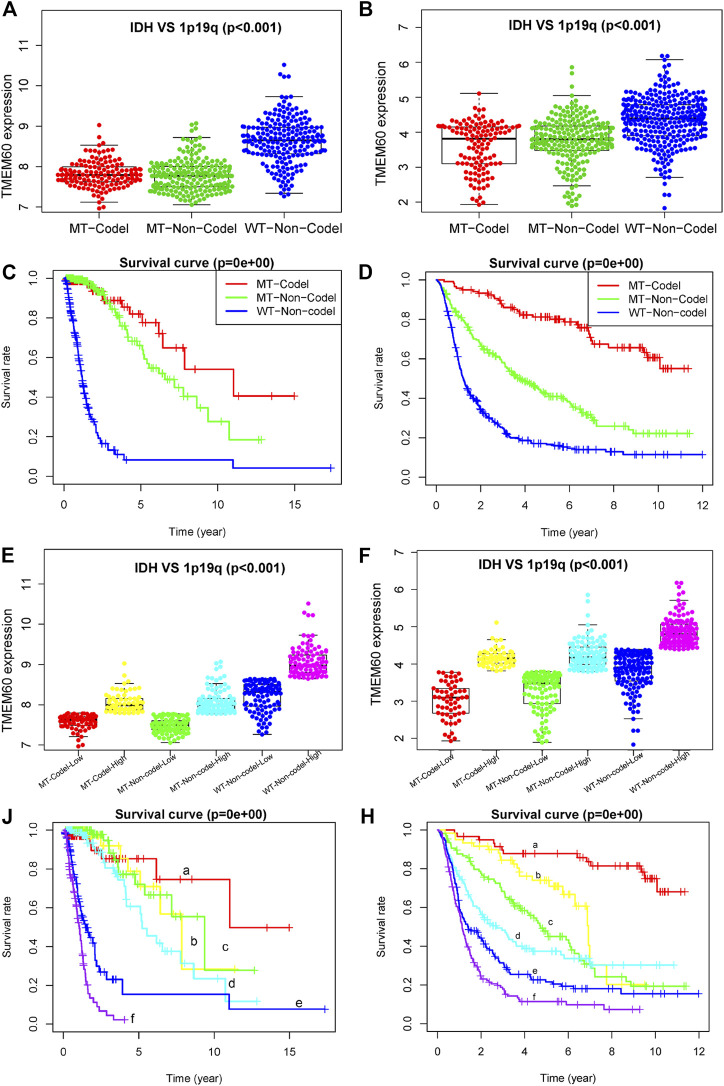
Influence of TMEM60, 1p19q status, and IDH status on the survival time of glioma patients. Expression level of TMEM60 in different subgroups of 1p19q status and IDH status in **(A)** TCGA and **(B)** CGGA. Survival curves of glioma patients in different subgroups of 1p19q status and IDH status in **(C)** TCGA and **(D)** CGGA. Six subgroups according to TMEM60 expression, 1p19q status, and IDH status in **(E)** TCGA and **(F)** CGGA. Survival curves of glioma patients in different subgroups of **(J)** TCGA and **(H)** CGGA. a, IDH MT-1p19q codel-low TMEM60: IDH mutant and 1p19q codeletion and TMEM60 low expression; b, IDH MT-1p19q codel-high TMEM60: IDH mutant and 1p19q codeletion and TMEM60 high expression; c, IDH MT-1p19q non-codel-low TMEM60: IDH mutant and 1p19q non-codeletion and TMEM60 low expression; d, IDH MT-1p19q non-codel-high TMEM60: IDH mutant and 1p19q non-codeletion and TMEM60 high expression; e, IDH WT-1p19q non-codel-low TMEM60: IDH wildtype and 1p19q non-codeletion and TMEM60 low expression; f, IDH WT-1p19q non-codel-high TMEM60: IDH wildtype and 1p19q non-codeletion and TMEM60 high expression.

The expression levels of TMEM60 was the lowest in the 1p19q codel-IDH mutant group, moderate in the 1p19q non-codel-IDH mutant group, and the highest in the 1p19q non-codel-IDH wild-type group of TCGA (Figure 6A; *p* < .001) and CGGA (Figure 6B; *p* < .001). Survival analysis in TCGA (Figure 6C; *p* < .001) and CGGA (Figure 6D; *p* < .001) revealed that the 1p19q codel-IDH mutant group had the longest survival time, followed by the 1p19q non-codel-IDH mutant group. The 1p19q non-codel-IDH wild-type group had the worst prognosis. We further divided the three groups into six subgroups in TCGA (Figure 6E) and CGGA (Figure 6F), according to the median value of TMEM60 expression. Survival analysis curves in TCGA (Figure 6J; *p* < .001) and CGGA (Figure 6H; *p* < .001) revealed that the overall survival time decreased in the following order: 1p19q codel, IDH mutant, and high TMEM60 expression levels; 1p19q codel, IDH wild-type, and high TMEM60 expression levels; 1p19q non-codel, IDH mutant, and low TMEM60 expression levels; 1p19q non-codel, IDH mutant, and high TMEM60 expression levels; 1p19q non-codel, IDH wild-type, and low TMEM60 expression levels; and 1p19q non-codel, IDH wild-type, and high TMEM60 expression levels.

### Correlation analysis between transmembrane protein 60 and immune cell infiltration

**FIGURE 7:**
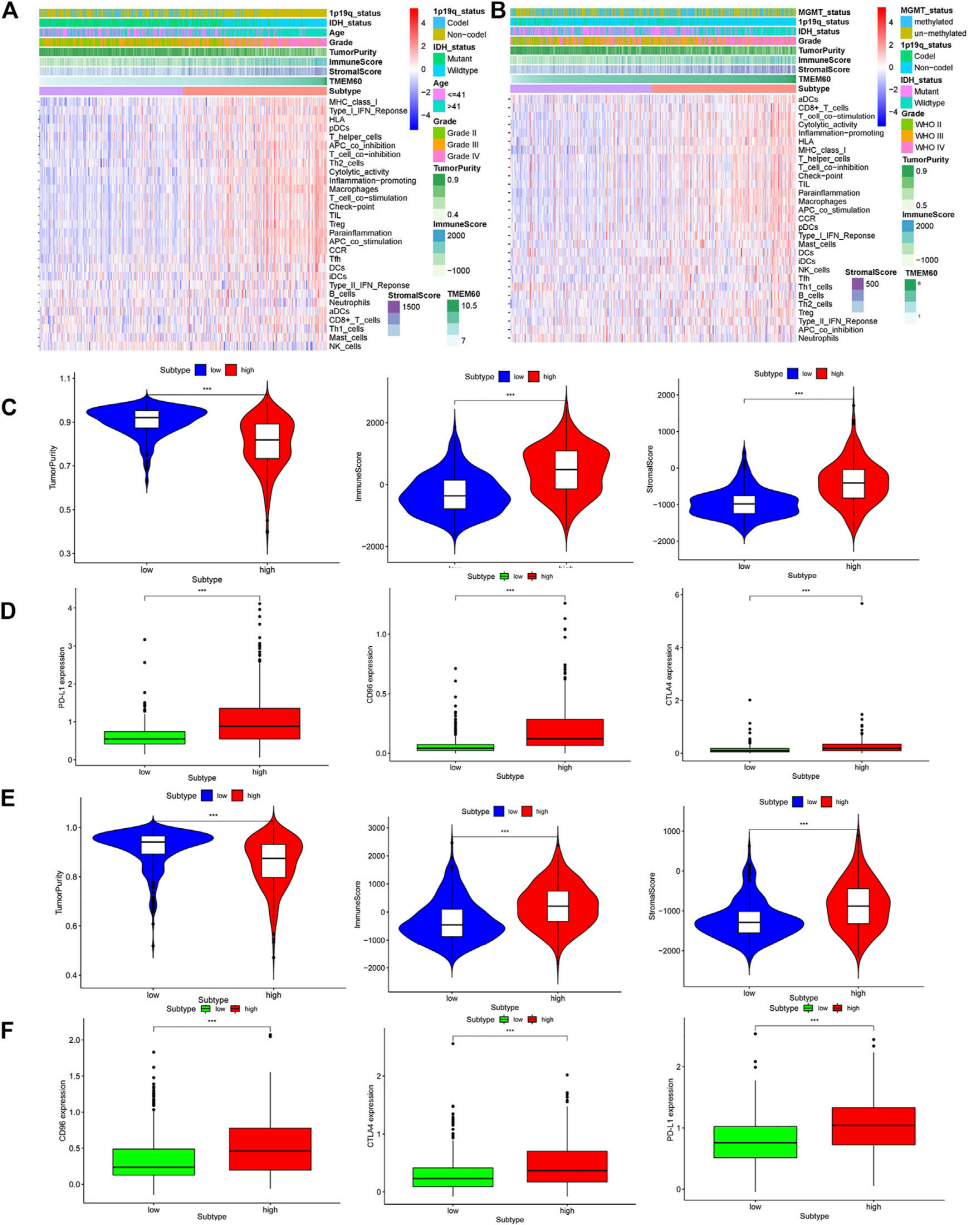
Relationship between TMEM60 and tumor microenvironment. Immune microenvironment heatmaps related to TMEM60 expression in **(A)** TCGA and **(B)** CGGA. Correlation between microenvironment-related indicators and TMEM60 in **(C)** TCGA and **(E)** CGGA. Correlation between TMEM60 and tumor immune checkpoint expression (CD96, PD-L1, CTLA4) in **(D)** TCGA and **(F)** CGGA.

**FIGURE 8:**
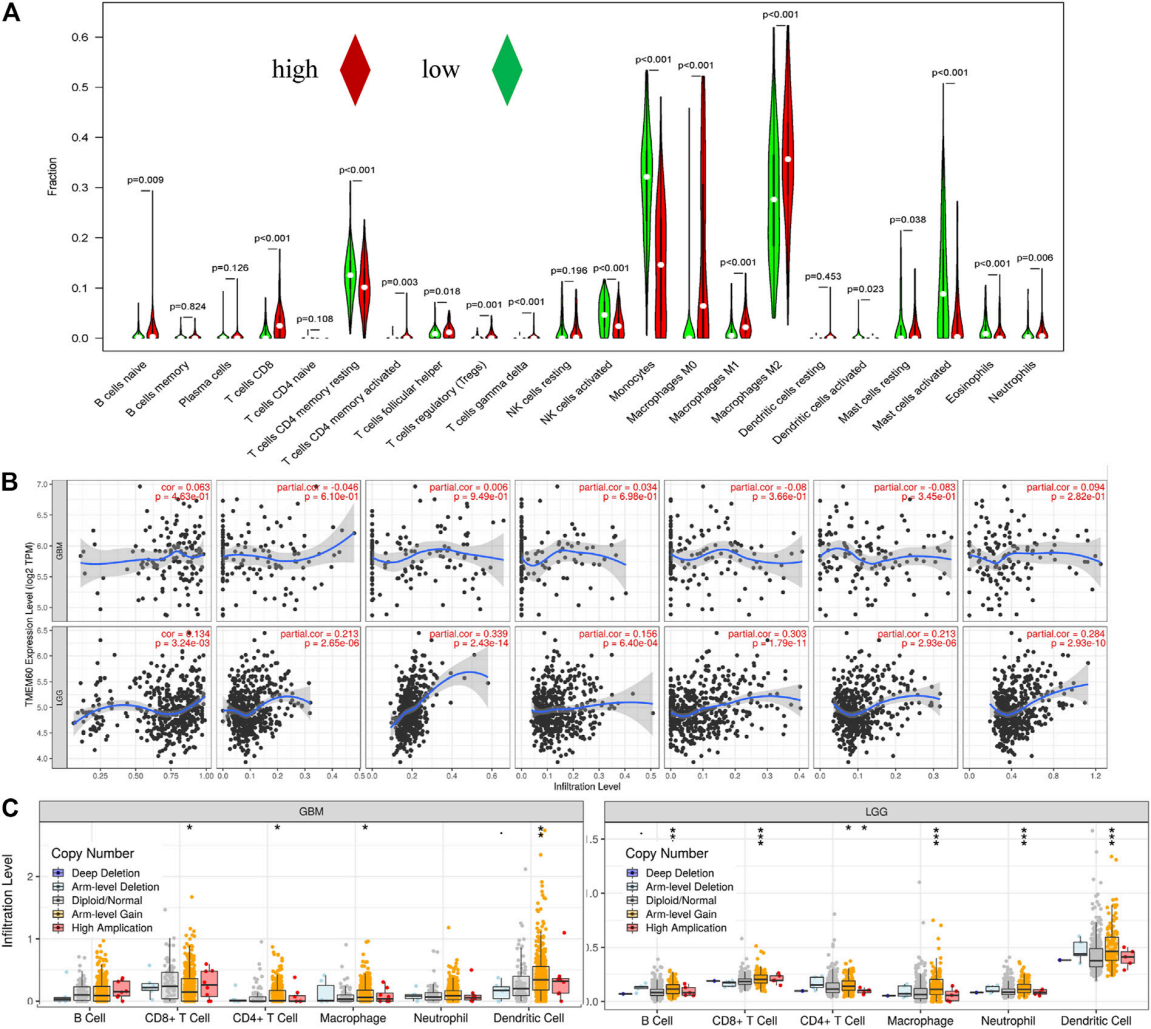
Correlation between TMEM60 and tumor immune cell infiltration. **(A)** Correlation between TMEM60 expression and immune cell infiltration in TCGA. **(B)** Correlation between TMEM60 expression and immune cell infiltration in the TIMRE database. **(C)** Infiltration levels among tumors with different somatic copy number alterations for TMEM60 in the TIMRE database.

We further explored the correlation between clinical characteristics and the immune microenvironment in TCGA (Figures 7A,C; *p* < .001) and CGGA (Figures 7B,E; *p* < .001). The clinical characteristics of patients in the high TMEM60 expression group were mainly 1p19q non-codel, IDH wild-type, >42 years of age, WHO Ⅲ–WHO Ⅳ grade, increased immune cells and stromal cells, decreased tumor purity, and increased activation of immune cells and pathways. We also analyzed the relationship between TMEM60 and immune checkpoints and found that patients with high TMEM60 expression levels in TCGA (Figure 7D; *p* < .001) and CGGA (Figure 7F; *p* < .001) also had high levels of CD96, PD-L1, and CTLA4. We further analyzed the infiltration of immune cells in the different TMEM60 expression groups of TCGA (Figure 8A) and CGGA (Supplementary Figure S1). The results showed that the infiltration of regulatory T-cells (Tregs), gamma delta T-cells, macrophages M0, neutrophils, and CD8^+^ T-cells increased, whereas that of CD4 memory resting T-cells, monocytes, and activated mast cells decreased. Based on the TIMRE database, the expression of TMEM60 was positively correlated with the number of cells infiltrated by CD8^+^ T-cells and macrophages in low-grade glioma (Figure 8B). The SCNA module provided a comparison of tumor infiltration levels among tumors with different somatic copy number alterations for TMEM60 deep deletion (−2), arm-level deletion (−1), diploid/normal (0), arm-level gain (Ostrom et al., 2020), and high amplification (Ostrom et al., 2021). The results showed that the copy number of TMEM60 was closely related to the number of infiltrated immune cells (Figure 8C).

### Functional enrichment analysis of differential genes

**FIGURE 9:**
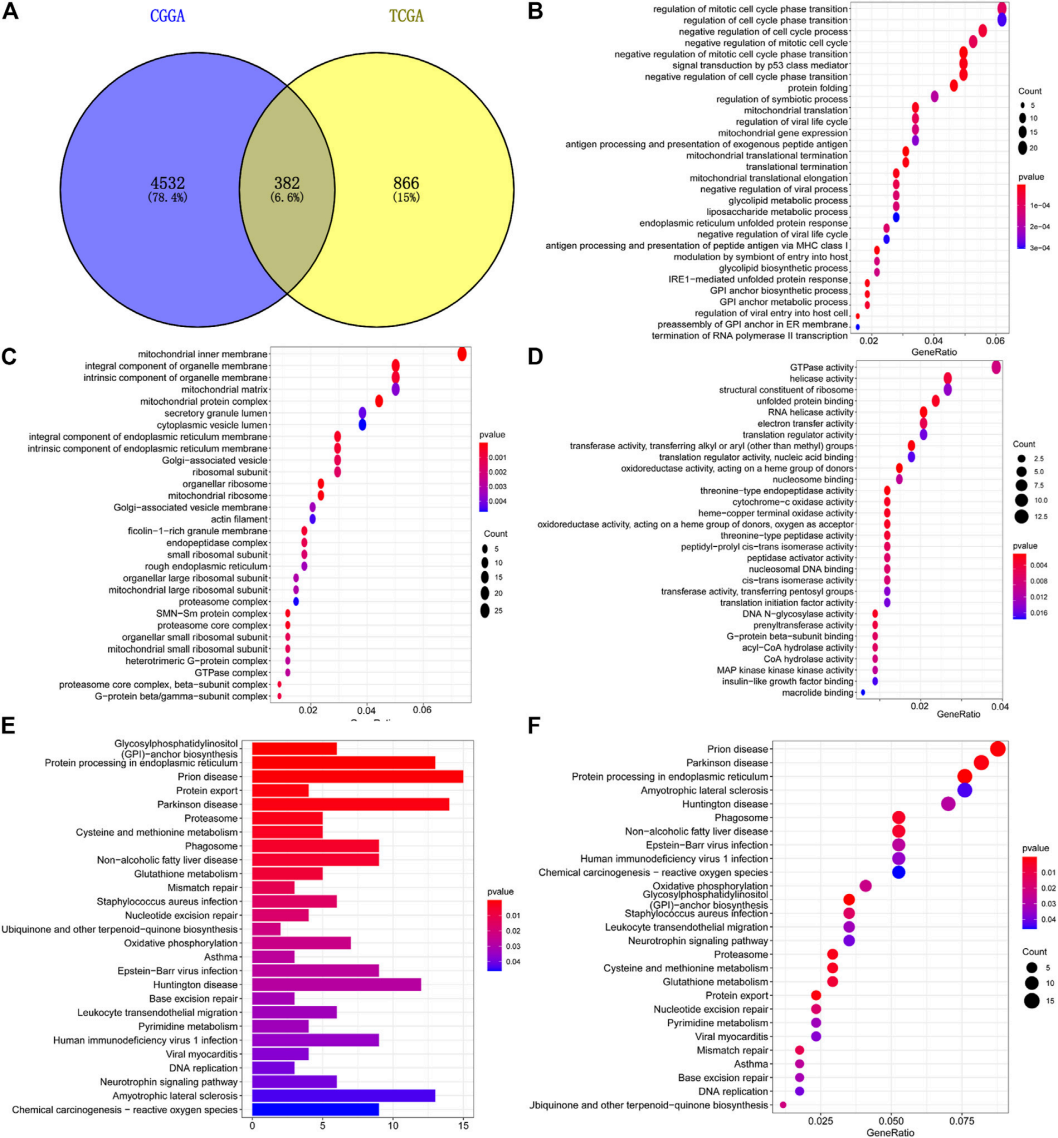
Enrichment analysis of co-expressed genes with TMEM60. **(A)** Intersection of TMEM60 co-expressed genes in TCGA and CGGA. **(B)** Cellular component analysis of co-expressed genes. **(C)** Biological process analysis of co-expressed genes. **(D)** Molecular function analysis of co-expressed genes. **(E,F)** Co-expressed gene enrichment pathways.

A total of 1,248 TMEM60-related genes in TCGA and 4,914 in CGGA were obtained with co-expression analysis (corFilter = .5). The intersection of these genes further revealed the existence of 382 genes (Figure 9A). GO analysis showed that the genes were enriched in biological process (neutrophil-mediated immunity; Figure 9B), cellular component (mitochondrial protein complexes, mitochondrial ribosomes; Figure 9C), and molecular function (catalytic activity, RNA, cell cycle, and DNA replication; Figure 9D). Kyoto Encyclopedia of Genes and Genomes (KEGG) analysis showed that the genes enriched were involved in the proteasome, DNA replication, lysosome, phagosome, mismatch repair, and cell cycle (Figures 9E,F).

### Silencing transmembrane protein 60 attenuates the proliferation ability of LN229 cells

**FIGURE 10:**
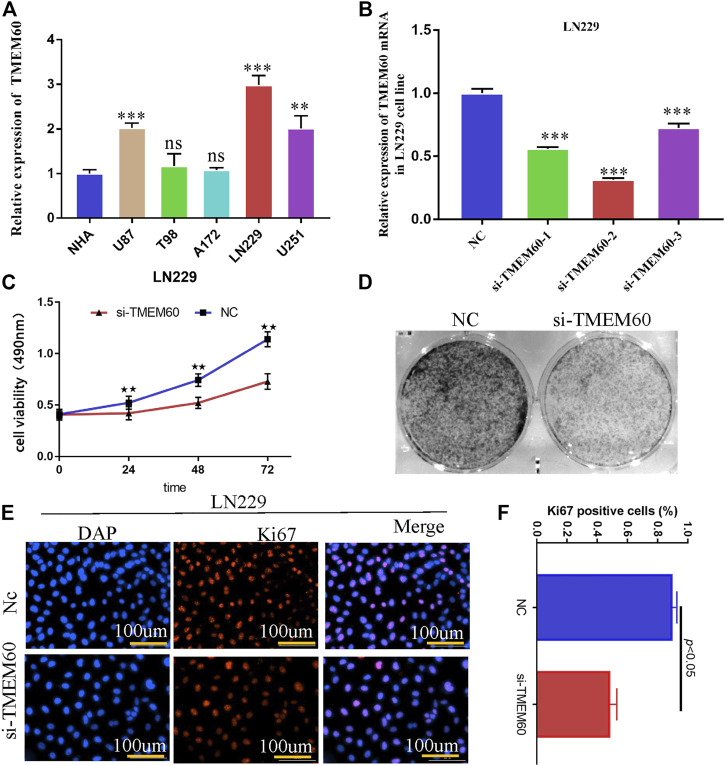
Silencing TMEM60 inhibits the viability and proliferation of glioma cells. **(A)** Relative expression of TMEM60 in various glioma and normal human astrocytes (NHA) cells. **(B)** Silencing efficiency of different TMEM60 inhibitors. **(C)** Activity of LN229 cells at different time points after TMEM60 silencing. **(D)** Clone formation ability of LN229 cells after TMEM60 silencing. **(E)** Expression of nuclear protein Ki67 in LN229 after TMEM60 silencing. **(F)** Quantification of nuclear protein Ki67 expression after TMEM60 silencing.

PCR experiments showed that the expression of TMEM60 in LN229, U251and U87 cell lines was significantly higher than that in normal astrocytes (Figure 10A). We selected LN229 as the research object and found that the silencing efficiency of si-TMEM60-2 at 72 h post-transfection was better than that of si-TMEM60-1 and si-TMEM60-3 at the same time point (Figure 10B). Results of the MTT assay also showed that the activity of LN229 cells gradually decreased at 0 h, 24 h, 48 h, and 72 h post-transfection (Figure 10C). We further found that si-TMEM60 significantly inhibited the colony formation of LN229 cells, compared with that in the negative control (Figure 10D), as well as the expression of the nuclear proliferation protein Ki67 (Figures 10E,F).

## Discussion

Gliomas are the most common primary malignant tumors of the central nervous system in adults. The annual incidence rate accounts for approximately 1.6% of systemic tumors, whereas the mortality rate accounts for 2.5% of systemic tumors (Bray et al., 2020). Numerous treatment methods, such as electric field therapy, nanotherapy, and immunotherapy, have been applied without any satisfactory results (Pereira et al., 2020). Cytogenetic and molecular genetic studies have shown that tumor development is a complex process involving multiple factors and stages (Mackay et al., 2017). Therefore, it is essential to identify molecular markers with high sensitivity to understand glioma diseases and improve diagnosis and treatment (Noushmehr et al., 2010).

We previously verified that TMEM60 is abnormally highly expressed in glioma and is related to the prognosis of glioma patients; thus, it can be used clinically as an independent prognostic and diagnostic factor. In the present study, univariate analysis results (HR = 3.97, 95% CI = 3.211–4.909, *p* < .001) showed that TMEM60 is a risk factor for glioma patients in TCGA, whereas multivariate analysis results (HR = 1.32, 95% CI = .935–1.868) were not statistically significant. Any inconsistencies could be attributed to differences in race, region, or clinical sample size. To draw a conclusion, we analyzed the expression of TMEM60 in 30 clinical samples and found that it is highly expressed in gliomas, and thus, it has a prognostic and diagnostic value.

To increase the uniformity of clinical results, WHO divides gliomas into five subtypes based on IDH mutations, 1p19q codel, and other molecular characteristics (Louis et al., 2016; Wesseling and Capper, 2018). We found that the TMEM60 expression level was higher in IDH wild-type patients than in IDH mutant patients and also in 1p19q non-codel patients than in 1p19q codel patients. Survival analysis revealed that 1p19q non-codel IDH wild-type patients with high TMEM60 expression levels had the worst prognosis. Therefore, the three genes (IDH, 1p19q, and TMEM60) could effectively distinguish different glioma subgroups and could be used as prognostic markers with potential clinical application value.

The tumor microenvironment mainly includes the extracellular matrix, soluble molecules, tumor stromal cells, secreted proteins, and RNAs (Balkwill et al., 2012; Catalano et al., 2013). Immune cells and stromal cells are the most common non-tumor cells, out of which the immune cells in brain tumors are mainly macrophages (Graeber et al., 2002). Macrophages can promote the malignant progression and treatment resistance of glioma cells by providing them with nutritional support (Yeo et al., 2022). We found that the clinical characteristics of patients with high TMEM60 expression levels were mainly IDH wild-type, 1p19q non-codel, WHO grade IV, >42 years of age, increased immune cells and stromal cells, decreased tumor purity, and increased activation of immune cells or pathways. Thus, the high TMEM60 expression group is in a highly immune state compared to the low expression group. In the two groups, infiltration of Tregs, gamma delta T-cells, macrophages, neutrophils, and CD8^+^ T-cells increased, whereas that of CD4 memory resting T-cells, monocytes, and activated mast cells decreased. Overexpression of immune checkpoint (CD96, PDL1, CTLA4) may lead to the depletion of anti-tumor immune cells in the microenvironment, which cannot play a good role in anti-tumor. Consequently, the abnormally high expression of TMEM60 affects the distribution of macrophages, Tregs, and other immune cells, increases tumor heterogeneity, and promotes tumor resistance to treatment, resulting in poor patient prognosis.

Wu et al. found that in U87 cell line, TMEM60 can affect the activity of glioma through akt, and silencing TMEM60 can inhibit the invasion, migration and proliferation of glioma cells (Wu et al., 2022). In LN229 cell line, we also proved that silencing TMEM60 can inhibit the proliferation of glioma cells. GO analysis of genes co-expressed with TMEM60 showed that they were enriched in processes such as neutrophil-mediated immunity, mitochondrial protein complex, mitochondrial ribosome, cell cycle, and DNA replication. KEGG analysis showed that they were enriched in processes involving proteasomes, DNA replication, lysosomes, phagosomes, mismatch repair, and cell cycle. Previously published studies suggested that the proteasome directly affects the renewal of misfolded proteins and of those that affect life activities, such as p53 and cyclin. The regulation of these proteins directly affects related biological functions, including cell cycle control (Mayer and Fujita, 2006), apoptosis (Hideshima et al., 2001), DNA repair, gene transcription, antigen presentation (Seliger et al., 2000; Kloetzel, 2001), signal transduction (Li et al., 2006), cancer (Vigneron and Van den Eynde, 2012; Chen et al., 2017b), inflammation, and immunity (Krüger et al., 2003). However, it is still unclear how TMEM60 regulates immune resistance to promote tumor malignant progress, although we analyze that it may be related to neutral mediated immunity. Whether the results of the *in vivo* experiment are consistent with those of the *in vitro* experiment remains to be further studied.

Overall, information from databases and clinical samples revealed that TMEM60 is a risk factor and could be used as a prognostic biomarker for glioma patients. Nevertheless, data from basic experiments were necessary to confirm the results. This is the first report on the abnormally high expression of TMEM60 in various glioma cell lines. In addition, we showed that suppression of TMEM60 expression significantly weakened cell proliferation and formation.

## Conclusion

In this study, we showed that TMEM60 combined with 1p19q and IDH has guiding significance for treating glioma. In addition, TMEM60 might participate in the process of tumor formation through the cell cycle and inhibit anti-tumor immunity by promoting the infiltration of Tregs and macrophages M0. Therefore, information from databases, clinical samples, and basic experiments revealed that TMEM60 is a new oncogene closely related to glioma patients’ prognosis with high potential for use as a therapeutic target.

## Data Availability

The datasets presented in this study can be found in online repositories. The names of the repository/repositories and accession number(s) can be found in the article/Supplementary Material.
