# Supplementary figures and images for: The new oncogene transmembrane protein 60 is a potential therapeutic target in glioma
Source: Front Genet. 2023 Jan 20;13:1029270. doi: 10.3389/fgene.2022.1029270 (PMC9895843; doi:10.3389/fgene.2022.1029270)

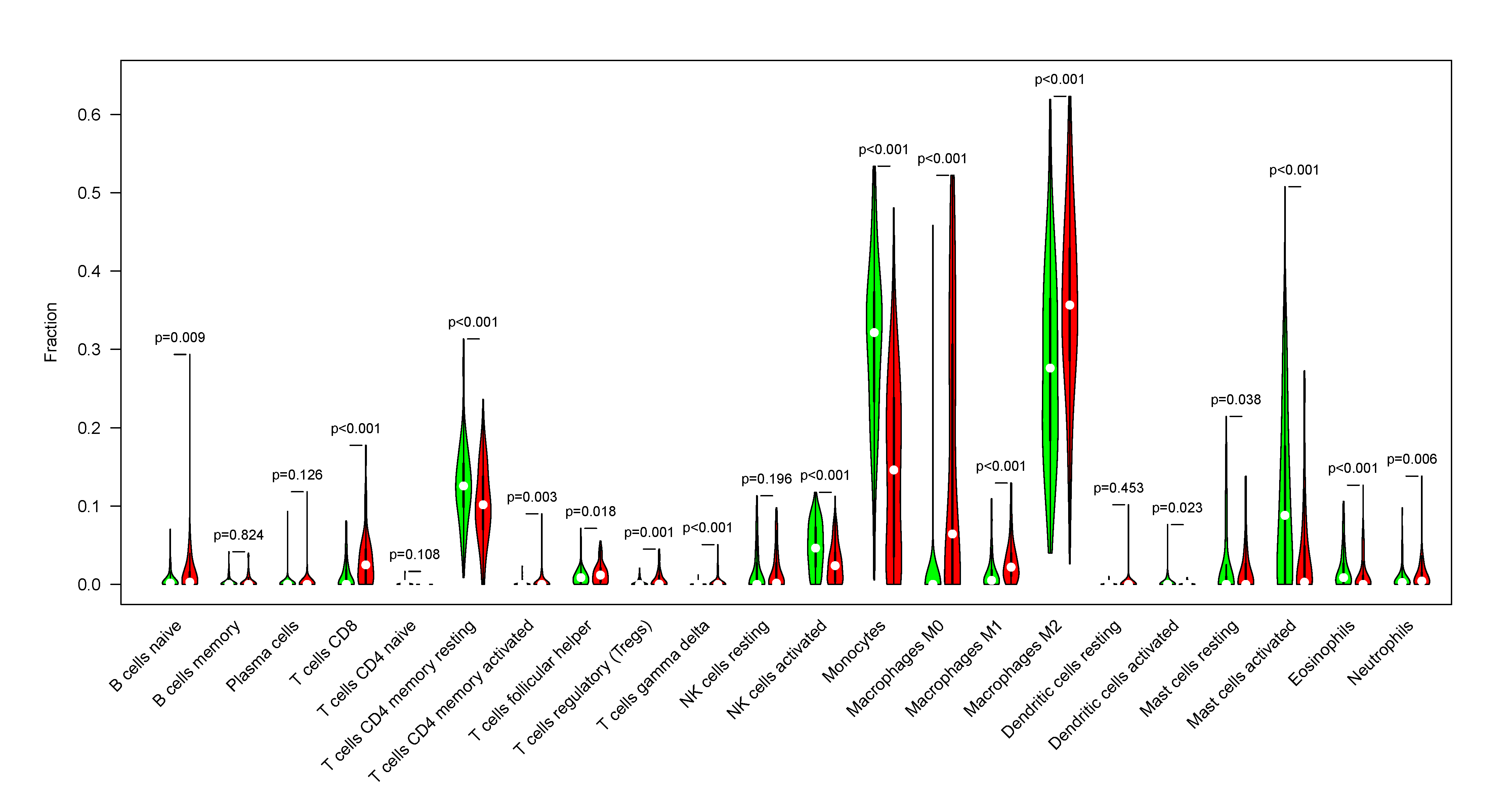

Supplement: Supplementary file 1 [file Image1.TIF]
